# DOF Enhanced via the Multi-Wavelength Method for the Moiré Fringe-Based Alignment

**DOI:** 10.3390/mi16030356

**Published:** 2025-03-20

**Authors:** Kairui Zhang, Haifeng Sun, Dajie Yu, Song Hu, Junbo Liu, Ji Zhou

**Affiliations:** 1 National Key Laboratory of Optical Field Manipulation Science and Technology, Chinese Academy of Sciences, Chengdu 610209, Chinaliujunbo@ioe.ac.cn (J.L.); 2 State Key Lab of Optical Technologies on Nano-Fabrication and Micro-Engineering, Chinese Academy of Sciences, Chengdu 610209, China; 3 Institute of Optics and Electronics, Chinese Academy of Sciences, Chengdu 610209, China; 4 University of Chinese Academy of Sciences, Beijing 100049, China

**Keywords:** lithography, alignment, Moiré fringe technology, multi wavelength measurement

## Abstract

Alignment systems are core subsystems of lithography, which directly affect the overlay accuracy of the lithography process. The Moiré fringe-based alignment method has the advantages of high precision and low complexity. However, the precision of this method is highly sensitive to variations in the gap between the wafer and the mask. To enhance the performance of Moiré fringe-based alignment, this paper proposes a novel method in which the multi-wavelength approach is used to enhance the imaging depth of focus (DOF). We use a multi-wavelength light to illuminate the alignment marks on the wafer and mask, which is combined with different sources. Then, we use the improved phase analysis algorithm to analyze the contrast of the Moiré fringe and calculate the Moiré fringe displacement. Experiments show that, in an alignment range of 1000 μm, the effective DOF can exceed 400 μm. It is evidenced that the accuracy of the Moiré fringe alignment is unaffected and remains at the nanometer level. Otherwise, with parameter optimization, the alignment DOF is expected to be further extended.

## 1. Introduction

In the integrated circuit (IC) industry, lithography is the mainstream equipment for large-scale chip production. The performance of lithography is generally evaluated based on three key factors: critical dimension (CD), throughput, and overlay accuracy. In IC manufacturing, producing a single chip requires multiple exposures, often tens or even hundreds of times. The overlay errors between layers can significantly impact the chip’s yield and performance [1]. Therefore, the accuracy of the lithography alignment system largely determines the magnitude of overlay errors, accounting for approximately 1/3 to 1/5 of the overall overlay precision [2]. With CDs now reaching the tens of nanometer range, this demands alignment accuracy at the nanometer or even sub-nanometer level [3].

Traditional alignment technologies can be classified into three types: geometric image-based alignment [4,5], intensity-based alignment [6,7], and phase-based alignment [8,9]. The alignment method based on geometric images is primarily used for early-stage lithography machines with lower accuracy. This method heavily relies on the image processing process and struggles to achieve clear simultaneous imaging of both the wafer and mask alignment marks on the CCD. The alignment method based on light intensity is one of the mainstream alignment methods today. This approach offers high precision but is sensitive to environmental factors, heavily dependent on the surface and edge characteristics of the alignment marks, and limited by the performance of the optical system. The phase-based alignment method is also widely used and can achieve high precision with relatively simple equipment. Since the discovery of the Moiré phenomenon by L. Raleigh in 1874 [10], Moiré fringes have found widespread applications in fields such as displacement, shape, and strain measurement. In recent years, the application of Moiré fringe technology in lithography optical alignment has gained increasing attention. Many research teams have focused on achieving precise alignment through Moiré fringe phase analysis, making significant progress in this field and achieving high-precision and high-sensitivity measurements [11,12]. In particular, the Moiré fringe alignment method based on overlapping gratings is very advantageous for wafer mask alignment due to its high accuracy and low equipment complexity. However, in practical applications, it is challenging to maintain a constant gap between the mask and the wafer throughout the alignment process due to the unevenness of the motion stage and wafer surface. This presents a significant challenge in calculating the Moiré fringe shift.

As lithography resolution improves, many new lithography techniques have emerged, such as nanoimprint lithography (NIL) [13,14] and electron beam lithography (EBL) [15], which offer advantages of high resolution and low cost in specific application areas. Compared to traditional lithography methods, NIL requires the mask to be in complete contact with the wafer for pattern transfer. As a result, there is a large variation in the gap between the mask and the wafer during the imprinting process. The Moiré fringe alignment method relies on detecting the interference signal formed by the superposition of two diffraction gratings with slightly different periods. The intensity and phase accuracy of the Moiré fringe signal collected at the receiving end can be easily affected by changes in the gap between the wafer and the mask. Moel et al. [16] introduced a coaxial interference alignment method, referred to as Coaxial Interference Alignment (OAI). This method uses a broad-spectrum light source to illuminate alignment markers, generating Moiré fringes for measuring alignment in multiple directions. It helps mitigate the impact of gap variations between the mask and the wafer on the fringe phase. However, the broad-spectrum light source has poor coherence, and after frequency-domain filtering, the contrast of the Moiré fringe signal becomes low, which increases the difficulty of phase analysis.

Based on the work and challenges addressed by the aforementioned teams, we propose a multi-wavelength controlled Moiré fringe alignment method. This method utilizes the principle that the Talbot self-imaging field positions differ when light sources of different wavelengths pass through a grating. Combining these diffraction fields effectively compensates for the dark field of a single wavelength, thereby generating a continuous intensity distribution. This method overcomes the issues of low contrast and shallow depth of field inherent in traditional Moiré fringes. Simulation and experimental results demonstrate the feasibility of this solution.

## 2. Alignment Principle

### 2.1. Moiré Fringe Alignment Theory

**Figure 1:**
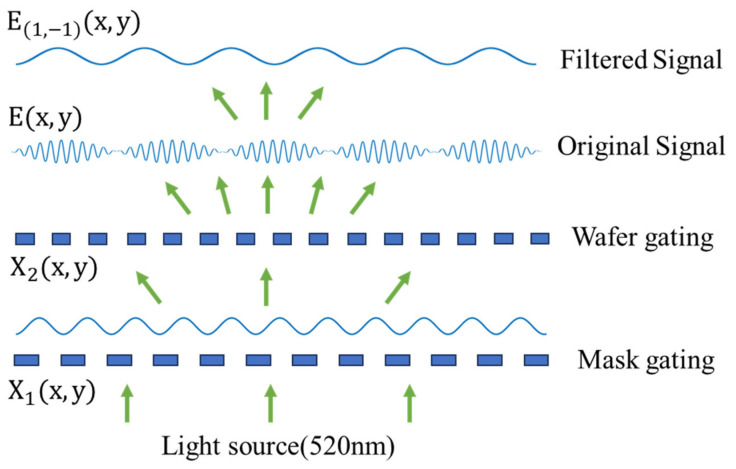
Schematic of dual grating superposition diffraction Moiré imaging.

The principle of Moiré’s fringe alignment method is to use a plane wave perpendicularly incident on two overlapping linear gratings with similar periods; the incident light is diffracted several times after modulation of the grating, and the harmonics of the diffracted wave are interfered and superimposed to form a Moiré’s fringe. As shown in Figure 1, the diffracted harmonics are superimposed and interfered to form the Moiré’s fringe field E(x,y). Plane-wave diffracting through the first grating is noted as m  and diffraction through the second grating is noted as n. Due to the influence of the numerical aperture (NA) of the receiving system, we only consider the case m+n=0, considering the effect of spectral intensity. Here, only the most distinguishable Moiré fringe diffraction fields from −1 to 1 level are calculated. $E_{\left(-1,1\right)}(x,y)$ can be written as:

(1) $$E_{\left(-1,1\right)}\left(x,y\right)=\sum_{-\infty }^{\infty }A_{n}B_{-n}\exp\{i2\pi n[f_{1}X_{1}\left(x,y\right)-f_{2}X_{2}\left(x,y\right)]\}$$

**Figure 2:**
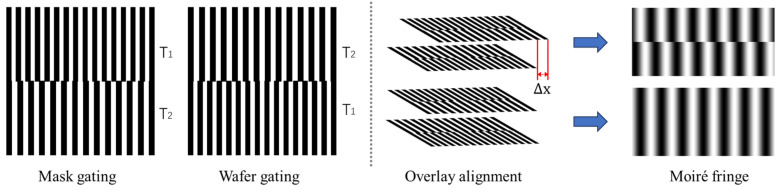
Schematic diagram of alignment marks and alignment process.

In Equation (1) $f_{1}=1/T_{1}$ and $f_{2}=1/T_{2}$ are the reference frequencies of the wafer and mask grating, $A_{n}$ and $B_{-n}$ are the Fourier transform coefficients, $X_{1}\left(x,y\right)$ and $X_{1}\left(x,y\right)$ are the distribution functions of the grating surface. When we use the alignment markers shown in Figure 2, the Moiré’s fringe generation period and grating period relationship can be written as:

(2) $$T_{m}=\frac{1}{f_{1}-f_{2}}=\frac{T_{1}T_{2}}{T_{2}-T_{1}}$$

where $T_{1}$ is the mask grating period and $T_{2}$ is the wafer grating period, the gratings with periods $T_{1}$ and $T_{2}$ will together form Moiré fringe with periods $\left|\frac{{T_{1}T}_{2}}{{T_{2}-T}_{1}}\right|$. The Moiré fringe amplifies the grating period by a factor of $\left|\frac{T_{1}}{{T_{2}-T}_{1}}\right|$ for precise alignment. When there exists an offset Δx between the two gratings, this change is reflected in the phase distribution of the Moiré fringe, which can be expressed in Equations (3) and (4):

(3) $$\Phi \left(x,y\right)=2\pi [f_{1}X_{1}\left(x,y\right)-f_{2}X_{2}\left(x,y\right)]$$

(4) $$\Phi \left(x+\Delta x,y\right)=2\pi [f_{1}X_{1}\left(x+\Delta x,y\right)-f_{2}X_{2}\left(x,y\right)]$$

The relationship of phase change and offset can be expressed as:

(5) $$\Delta x=\frac{\Delta \Phi }{2\pi }\frac{1}{f_{1}+f_{2}}=\frac{\Delta \Phi }{2\pi }\frac{T_{m}}{2}$$

### 2.2. Depth of Focus in the Talbot Effect (Focused Depth Theory in the Talbot Effect)

**Figure 3:**
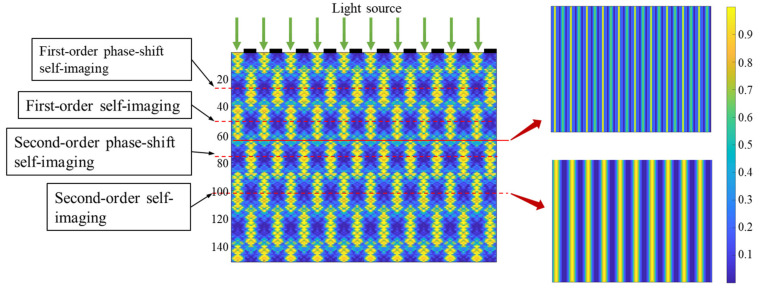
Grating self-image light field distribution.

According to the scalar diffraction theory, monochromatic plane wave incident perpendicularly on a periodic grating and give rise to a periodic self-imaging effect, which is known as the Talbot effect and was first proposed by Henry Fox Talbot in 1836 [17]. Specifically, a monochromatic plane wave incident perpendicularly on a periodic object along the direction of propagation of the light field produces a self-image of the object with a period of $Z=2nT^{2}/\lambda ,(n=1,2,3\ldots \ldots )$, where n is a positive integer and T is the mask period, λ is the wavelength. A self-image with π-phase shift will be formed at $Z=(2n-1)T^{2}/\lambda ,(n=1,2,3\ldots \ldots )$, which is a diffractive image, not a lens image, and its imaging resolution is very high, close to the half-wavelength diffraction limit. In addition, at the junction of the self-image and the π-phase-shifted self-image, a weak frequency doubling of the light intensity occurs, referred to as the “frequency doubling effect”, as shown in Figure 3.

When the Talbot effect and the frequency doubling effect are applied in the field of lithographic alignment, the depth of focus (DOF) of the alignment system can be extended to a certain extent, and it achieves a small-period imaging effect using a large-period grating. The depth of focus (DOF) is defined as the interval in which the light can be clearly imaged along the direction of light propagation, and it usually refers to the range in which the image plane can be moved when the change in the wave aberration of the system caused by the movement of the system image plane is not more than a quarter of the wavelength. The depth of focus is inversely proportional to the magnification, and usually, the larger the magnification, the shorter the depth of focus, so the method based on Moiré’s fringe alignment is limited by the Talbot effect, which requires strict control of the gap between the mask and the wafer. Therefore, the effective focal depth length is an important factor affecting the adaptability of the alignment system.

According to the self-imaging theory of the Talbot effect, the ideal self-imaging surface can be approximated in a form similar to the wave aberration in an optical imaging system as $z_{s}=2nT^{2}/\lambda ,(n=0,1,2,3\ldots \ldots )$ This self-imaging effect produces wavefront aberrations in the image plane, which, in turn, leads to graphical distortion. This wavefront distortion is due to the combined effect of the diffraction levels of all the different wave vectors. The diffracted light of level 0 is used as a reference:

(6) $$\delta_{n}=Z_{M}-Z_{M}cos\theta_{n}$$

The wavefront distortion introduced by all diffraction levels can be expressed as:

(7) $$\Delta \Psi =Z_{M}\sum_{n=1}^{M}\delta_{n}=Z_{M}\sum_{n=1}^{M}\left(1-cos\theta_{n}\right)$$

where M is the maximum diffraction level, while, in the traditional definition, the depth of focus is such that the wavefront aberration due to defocusing cannot exceed, so the effective interval of the depth of focus can be written as:

(8) $$DOF=Z_{M}=\frac{\lambda }{4\sum_{n=1}^{M}\left(1-cos\theta_{n}\right)}$$

Bringing the diffraction equation $Tsin\theta_{n}=n\lambda$ into Equation (8) yields that:

(9) $$DOF=Z_{M}=\frac{\lambda }{4}\frac{1}{\sum_{n=1}^{M}1-\sqrt{1-{\left(\frac{n\lambda }{T}\right)}^{2}}}=\frac{T^{2}[1+\sqrt{1-{\left(\frac{n\lambda }{P}\right)}^{2}}]}{4\sum_{n=1}^{M}n^{2}\lambda }$$

**Figure 4:**
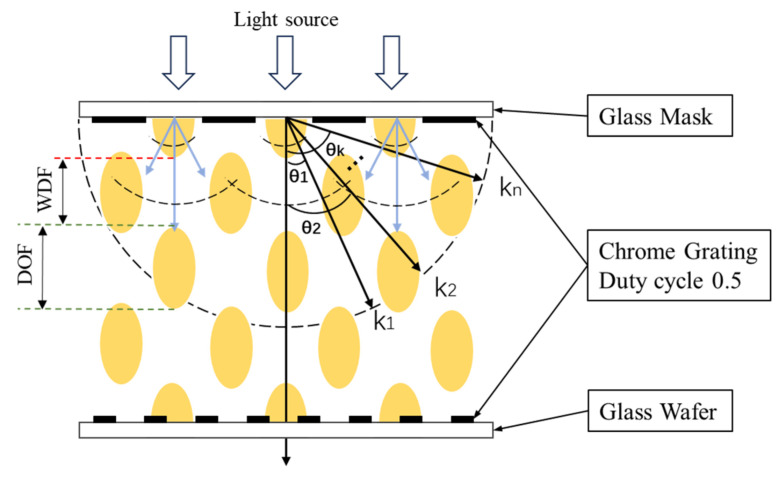
Frequency Doubling Effect Schematic.

At a single wavelength, the DOF and WDF regions alternate with a specific period after the mask grating, as shown in Figure 4.

By introducing multi-wavelength incidence, since different wavelengths have different self-imaging positions, the superposition of these self-imaging intervals can result in a continuous frequency-doubling region. This approach allows a large-period grating to achieve the imaging effect of a small-period structure, extends the depth of focus, and also has a corrective effect on minor defects in periodic micro-nanostructures. Theoretically introducing multiple beams of non-correlated narrowband light sources and the effect of the transmission distance of z-direction light will be taken into account, Equation (1) can be rewritten, and the distribution of the light intensity of the interference fringes in the grating-modulated Moiré’s fringe diffraction field can be expressed as:

(10) $$I\left(x,y,z\right)=\sum_{n=1}^{n}\sum_{\lambda_{n\;min}}^{\lambda_{n\;max}}Q\left(\lambda \right)*{\left|E\left(x,y,z\right)\right|}^{2}\;=\sum_{n=1}^{n}\sum_{\lambda_{n\;min}}^{\lambda_{n\;max}}Q\left(\lambda \right)*\left\{\sum_{m}a\left(x,y,z\right)+b\left(x,y,z\right)cos\left(\Phi \left(x,y\right)+2\pi \left(f_{x}x+f_{y}y\right)\right)+n(x,y,z)\right\}$$

where $a\left(x,y,z\right)$ denotes the background light intensity, $b\left(x,y,z\right)$ denotes the amplitude intensity, n(x,y,z) denotes the noise intensity, and $f_{x}$ and $f_{y}$ denote the components of the spatial frequency in the x and y directions. Where n represents the number of uncorrelated narrowband light sources and $\lambda_{max}$ and $\lambda_{min}$ represent the upper and lower bounds of the bandwidth of the nth beam.

Therefore, the width of the dark field (WDF) can be expressed as:

(11) $$WDF=N(\frac{2T^{2}}{\lambda_{min}}-\frac{2T^{2}}{\lambda_{max}})$$

where N denotes the Talbot distance multiplier along the direction of light propagation, which can be an integer or a fraction, and different wavelengths of incidence will cause the DOF and WDF to fill each other to form a continuous doubling interval.

## 3. Depth of Focus and Contrast Analysis

During the whole process of lithography alignment, the distance between the mask and the wafer will inevitably change at the micrometer level, so this requires that the calculated results of the images we acquire at different positions remain the same. In practice, due to the influence of the Talbot effect CCD camera acquired Moiré’s fringes with the change of grating spacing and periodic out-of-focus phenomenon; this phenomenon is related to the grating period and illumination wavelength.

In fact, Moiré’s fringes and the Talbot effect both involve the case of plane waves perpendicularly illuminating a periodic object. In the traditional methods of Moiré’s fringes alignment, in order to obtain the best contrast, the distance d between two gratings is required to be a positive integer multiple of the Talbot distance, i.e., $Z=nT^{2}/\lambda ,(n=1,2,3\ldots \ldots )$, this greatly increases the difficulty of engineering applications. Introducing a multi-wavelength narrow-bandwidth light source, as described in the previous section, the superposition of diffracted fields of different wavelengths can result in a continuous octave interval with clear contrast, which can significantly increase the process adaptability of the alignment system.

**Figure 5:**
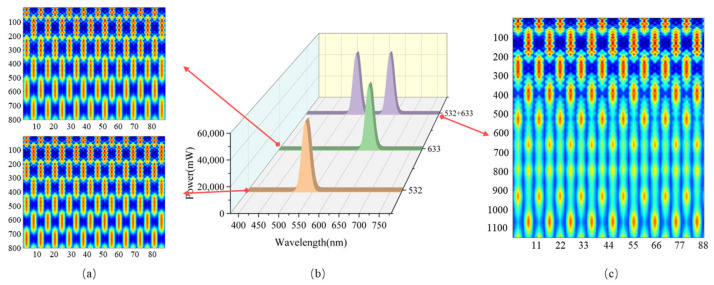
Spectral curves at different wavelengths and Talbot self-imaging light field distributions. (**a**) Talbot self-imaging intensity distribution behind the grating with 8.8 μm grating modulation under incident light of 663 nm and 532 nm wavelengths, from top to bottom. (**b**) The spectrum of 532 nm, 633 nm, and the combined beam of 532 nm and 633 nm, from bottom to top. (**c**) The intensity distribution of Talbot self-imaging behind the grating under 8.8 μm grating modulation with the combined incident light of 532 nm and 633 nm.

Figure 5a presents the Talbot effect intensity distribution after an 8.8 μm-period grating illuminated by light sources of different wavelengths. It can be observed that under 532 nm illumination, self-images without phase shifts appear at distances of 291.12 μm, 582.25 μm, etc., from the grating. These positions correspond to the optimal alignment points for traditional Moiré fringe alignment. When the gap between the wafer and the mask falls within this range, it defines the depth of focus (DOF) for alignment, ensuring the highest contrast and the smallest phase unwrapping error. At distances of 145.56 μm and 436.69 μm, self-images with a π-phase shift appear. If the gap between the mask and the wafer is at these positions, the contrast decreases, and the phase unwrapping error increases. Under 633 nm illumination, the DOF positions are at 244.67 μm and 489.35 μm from the grating. When 532 nm and 633 nm light sources are combined and used to illuminate the alignment marks, the periodic self-imaging distances for different wavelengths vary. As a result, their intensity distributions overlap to form an extended continuous DOF region. Within this region, the alignment grating on the mask undergoes a frequency-doubling effect, interfering with the alignment grating on the wafer to generate Moiré fringe signals. The contrast of these Moiré fringes is significantly enhanced.

Figure 5b presents the spectral curve used in the simulation, where the vertical axis represents energy and the horizontal axis represents wavelength. Figure 5c shows the Talbot effect intensity distribution when 532 nm and 633 nm light sources, each with a 10 nm half-bandwidth, simultaneously illuminate an 8.8 μm-period grating. It can be observed that under multi-wavelength illumination, starting from 450 μm, the self-imaging regions of different wavelengths overlap, forming a continuous bright region of approximately 450 μm in length. As long as the mask-to-wafer gap falls within this bright region, a high-contrast Moiré fringe image can be obtained.

**Figure 6:**
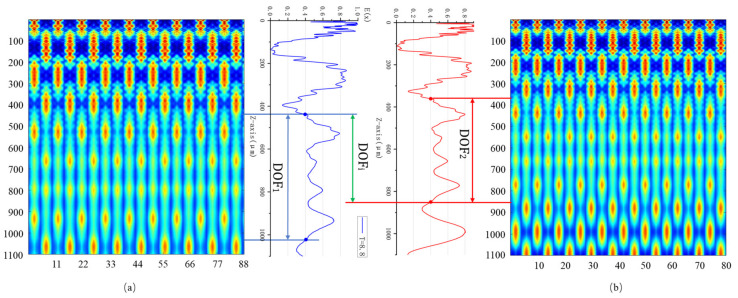
Talbot effect light intensity distribution at different grating periods and z-direction normalized light intensity distribution curves: (**a**) grating period of 8 μm (**b**) grating period of 8.8 μm.

Moiré fringe alignment markers commonly use two sets of gratings with slight period differences, and the Talbot self-imaging effect is jointly influenced by the light source wavelength *λ* and the grating period *T*. Therefore, both the chosen light source wavelength and grating period must be carefully considered during the alignment process. When aligning using gratings with periods T=8 μm and T=8.8 μm, the starting and ending points of the extended DOF regions in the *z*-direction differ due to the variation in grating periods. Figure 6 shows the extended DOF range for different grating periods under the same incident wavelength, as well as the normalized intensity distribution curves across the entire depth of focus range.

Let the DOF corresponding to *T* = 8 μm be denoted as *DOF*_1_ and that of T=8.8 μm as *DOF*_2_. The continuous DOF region available for alignment is given by the intersection of *DOF*_1_ and *DOF*_2_, denoted as *DOF_i_*. The figure presents the simulated Talbot self-imaging intensity distribution along the z-direction for different grating periods. Due to the excessively high intensity in the half-transmissive region immediately behind the grating ridges, the normalized self-imaging field appears generally weaker. However, in practice, a normalized intensity of 0.4 is sufficient to produce clear fringe images. Here, we define the DOF region as the range where the normalized intensity is I≥0.4. *DOF*₁ corresponds to the grating period T=8.8 μm, with a range [439 μm, 1023 μm], *DOF*₂ corresponds to the grating period *T* = 8 μm, with a range [362 μm, 850 μm]. The intersection *DOFᵢ* is the overlapping region of *DOF*₁ and *DOF*_2_. $DOF_{i}=DOF_{1}\cap DOF_{2}=(439,1023)\cap (362,850)=(439,850)$. As long as the mask-to-wafer gap remains within this range during the entire alignment process, a clear Moiré fringe pattern can be obtained.

**Figure 7:**
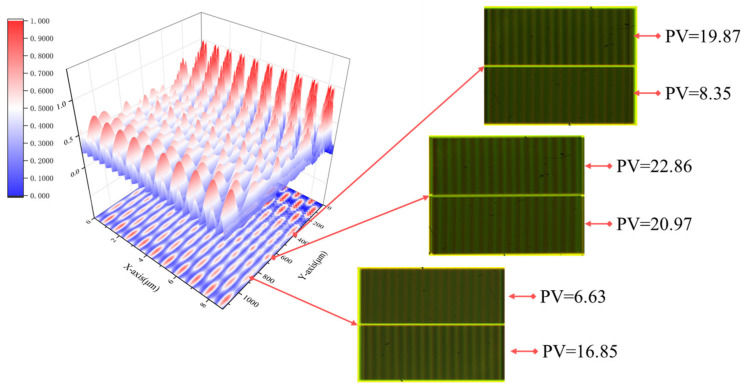
3D plots of the Talbot effect light intensity distribution. The three images on the right are experimental Moiré fringe patterns captured by the CCD at different distances along the z−axis. The corresponding average peak−to−valley (PV) value for each row is provided on the right side of each image.

Figure 7 presents the 3D intensity distribution along the z-direction when both 532 nm and 633 nm light sources illuminate gratings with periods of 8 μm and 8.8 μm. Additionally, experimental images of Moiré fringes captured by the CCD at different positions are provided. In the 3D intensity distribution, each column represents the normalized sum of the intensities at the same location for both the 8 μm and 8.8 μm period gratings. The figure shows that the first continuous and distinct frequency-doubling region appears at 362 μm for the 8 μm grating. However, between 362 μm and 439 μm, the 8.8 μm grating does not form a continuous DOF, resulting in Moiré fringes with high contrast in the upper part but blurred fringes in the lower part of the CCD image. In the range of 439 μm to 850 μm, both the 8 μm and 8.8 μm gratings exhibit a continuous DOF, leading to high-contrast Moiré fringes across both the upper and lower parts of the alignment mark image captured by the CCD. Beyond 850 μm and up to 1023 μm, the DOF for the 8.8 μm grating continues, while the DOF for the 8 μm grating ends at 850 μm. As a result, the Moiré fringes in the CCD image show poor contrast in the upper part but clear fringes in the lower part. For a more objective representation of contrast differences, the average peak-to-valley (PV) value for each row is displayed on the right side of each experimental image.

In summary, the earliest appearance of the continuous DOF region is at 362 μm, and in the range of 439 μm to 850 μm, the frequency-doubling effect of the two gratings with different periods is highly pronounced. Within this region, clear Moiré fringe alignment images can be captured. Beyond 850 μm and up to 1023 μm, the intensity of the continuous DOF region gradually weakens and eventually disappears.

The reason for this phenomenon has been discussed in the previous section. The Talbot self-imaging distance follows a fixed proportional relationship with the incident wavelength and the grating period, similar to a cosine function. When two beams of different wavelengths are incident, their intensity superposition behaves like the summation of two cosine functions with different periods. As a result, the DOF starts at half the least common multiple (LCM) of the two cosine function periods and ends at the LCM itself. From the simulation results, it can be seen that with the combination of two wavelengths, the variation range of the gap between the mask and the wafer can be expanded to approximately 3 to 4 times the original range. If more wavelengths are introduced, assuming the grating period remains the same and light intensity attenuation is not considered, the theoretically available DOF can reach half of the entire range.

In contrast, when simulating with a 10 nm half-bandwidth light source, the filling of different wavelength components smooths out the Talbot imaging edges. While some fine details are sacrificed, this approach ensures a more uniform DOF region over a larger range, effectively relaxing the strict requirement for maintaining a constant mask-to-wafer distance during lithographic alignment.

## 4. Experiment and Discussion

**Figure 8:**
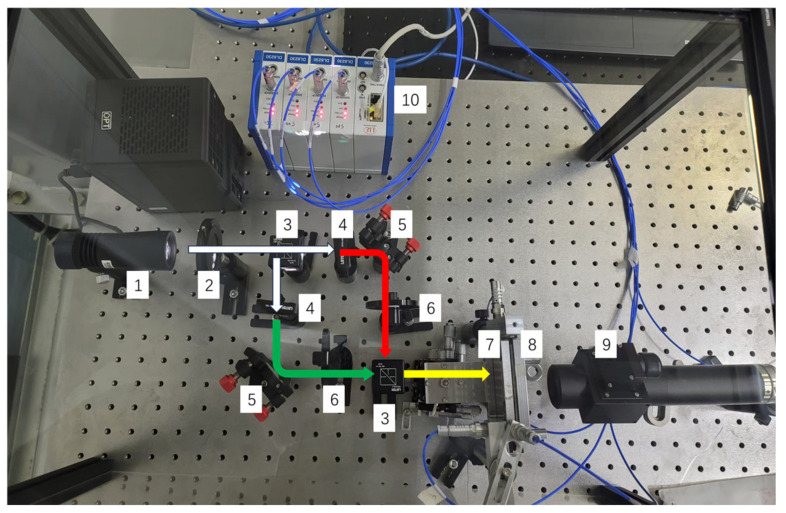
Experimental setup diagram. 1. light source; 2. diaphragm; 3. beam splitter; 4. optical filter; 5. reflector; 6. adjustable attenuator; 7. mask; 8. wafer; 9. CCD; 10. capacitive sensor.

The experimental setup is shown in Figure 8. The alignment marks on the mask are mounted on a manually adjustable stage with three translational degrees of freedom (XYZ) and one rotational degree of freedom (Rz). The alignment marks on the wafer are fixed on a holder, while the CCD camera is equipped with a 10× magnification lens and connected to a computer for acquiring and displaying Moiré fringe images. Light from an LED source (wavelength range 430 nm to 750 nm) is split into two paths using a beamsplitter. The first path passes through a bandpass filter to isolate green light at 532 nm with a bandwidth of 10 nm, while the second path passes through another bandpass filter to isolate red light at 633 nm with a bandwidth of 10 nm. The two beams of light are combined after passing through mirrors and a beam splitter and finally converge to illuminate the alignment marks of the wafer and the mask. The reflected light is collected by an objective lens and imaged onto a CCD camera (MER2-2000-19U3C, resolution: 5496 × 3672 pixels, pixel size: 2.4 × 2.4 µm^2^), forming Moiré fringes.

**Figure 9:**
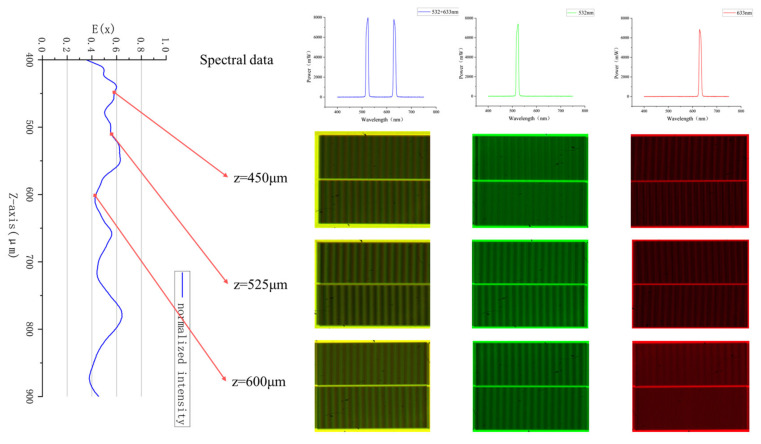
The experimental Moiré fringe images captured at the same distance under different wavelength light sources. On the left is a normalized line plot of the light intensity distribution on the same plane for gratings with T = 8 μm and T = 8.8 μm. The image with a yellow background was captured under multi-wavelength illumination. The green image was captured under 532 nm illumination, and the red image was captured under 633 nm illumination.

In Figure 9, we present Moiré fringe images captured by the CCD under three different alignment light sources with varying gap distances between the mask and the wafer. Since the white light source used in the experiment does not have a uniform energy distribution across the 430 nm to 750 nm range, the energy of the light beams after filtering is different. A variable attenuator is used to adjust the energy levels of the 532 nm and 633 nm light to the same level. From the figure, it is intuitively clear that the Moiré fringe contrast varies under different light sources at the same position. In the previous section, it was stated that light sources with wavelengths of 532 nm and 633 nm were combined and then illuminated onto gratings with T = 8 μm and T = 8.8 μm, resulting in an effective depth of focus ${\mathrm{DOF}}_{i}=(439,850)$. Therefore, we used the combined light source to illuminate the alignment marks and captured Moiré fringe images at 450 μm, 525 μm, and 600 μm. When only the 532 nm green light is used as the alignment light source, a clearer Moiré fringe image can be obtained at 600 μm, while the contrast at 450 μm is noticeably reduced. In contrast, with the 633 nm red light, the Moiré fringe contrast is weaker at 600 μm and clearer at 450 μm. Since the 525 μm position falls within the DOF range of both the 532 nm and 633 nm light sources after aligning with the silicon wafer marks, clear Moiré fringe images are obtained for both wavelengths at this position. This aligns with the theoretical and simulation results mentioned earlier, proving that using a combined multi-wavelength light source as the alignment light can effectively extend the depth of focus of the Moiré fringe image, thus increasing the process adaptability of the alignment system.

To more intuitively represent the differences in imaging effects at different distances, we introduce several image evaluation metrics, such as the Weber contrast for simple images [18], the Michelson contrast [19], and the root mean square (RMS) contrast [20]. The Weber contrast is suitable for situations where the target is small and located in a large uniform background, while the Michelson contrast is used to calculate the contrast of images with periodic bright and dark distributions, such as sinusoidal fringes. The RMS contrast evaluates the overall intensity fluctuations in the image, reflecting the uniformity of the fringes. Their formulas can be expressed as follows:

(12) $$C_{Weber}=\frac{I-I_{b}}{I_{b}}$$

(13) $$C_{M}=\frac{I_{max}-I_{min}}{I_{max}+I_{min}}$$

(14) $$C_{RMS}=\sqrt{\frac{1}{N}\sum_{i=1}^{N}{\left(I_{i}-\bar{I}\right)}^{2}}$$

In Equation (12), *I* represents the background brightness, and $I_{b}$ is the brightness difference between the target and the background. Weber contrast is typically used in situations where the background brightness exceeds the target brightness, particularly suited for dark targets (such as dark spots in optical experiments) displayed against a bright background.

In Equation (13), *I*_*max*_ and *I*_*min*_ represent the maximum and minimum light intensities in the image, respectively. Michelson contrast is applied in cases where the bright/dark distribution is relatively symmetric. It plays an important role in fields such as optical interference patterns, fringe analysis, and image quality assessment.

In Equation (14), *N* is the total number of pixels, *I*_*i*_ is the brightness of the *i*-th pixel, and $\bar{I}$ is the average brightness. Root mean square (RMS) contrast can measure the intensity of brightness fluctuations across the entire image and is suitable for contrast analysis of interference fringes under low-light conditions. These contrast metrics help to objectively assess the quality of Moiré fringe images, especially in different imaging conditions.

In Table 1, we introduce three evaluation metrics, $C_{Weber}{,\;C}_{M},\;C_{RMS},$ to assess the contrast of Moiré fringe images captured under different wavelengths at the same distance. The best-performing data in the table are underlined, while the second-best data are shown in bold.

From the data, it can be observed that at 450 μm, the 532 nm green light falls within the WDF (weak depth of focus) region, resulting in poor image contrast, whereas the 633 nm red light falls within the DOF (depth of focus) region, leading to better contrast. The combined multi-wavelength light source enhances contrast by superimposing the diffraction intensities of 532 nm and 633 nm, achieving the best contrast performance, as reflected in three evaluation metrics.

Similarly, at 600 μm, the 532 nm green light falls within the DOF region and exhibits good contrast, while the 633 nm red light falls within the WDF region, leading to weaker contrast. Again, the combined light source performs best, excelling in all three contrast evaluation metrics. This objectively validates the scientific basis and feasibility of multi-wavelength beam combination for depth of focus extension and contrast enhancement.

**Figure 10:**
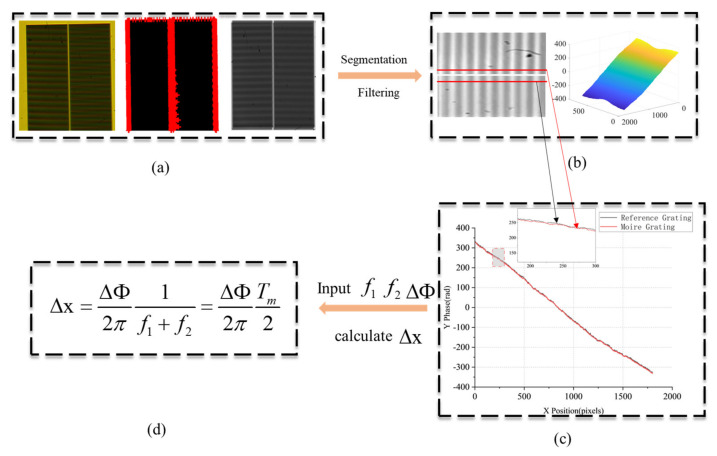
Algorithm Implementation Flow: (**a**) Moiré fringe pattern after pre-processing. (**b**) The filtered Moiré fringe images and the phase unwrapping distribution maps. (**c**) One of the rows of phase distribution curves with phase difference (**d**). The key parameters are obtained to calculate the offset from Equation (5).

To verify the feasibility and accuracy improvement of this method, we analyze and process the Moiré fringe images obtained under multi-wavelength illumination after beam combination, and the specific process is shown in Figure 10.

Step_1: To enhance the accuracy of the phase analysis algorithm, the captured images undergo a series of pre-processing operations, including grayscale conversion, image dilation, binarization for edge point selection, and rotation correction to align the image properly. These steps aim to reduce noise and eliminate errors caused by CCD installation angle misalignment.

Step_2: After pre-processing, the two grating regions in the image are segmented and processed separately using a two-dimensional Fourier transform. A Hanning window is applied for filtering to remove high-frequency noise, followed by median filtering to eliminate salt-and-pepper noise [21]. This results in two wrapped phase distribution matrices.

Step_3: The two wrapped phase distribution images are then unwrapped, and a pixel-by-pixel subtraction is performed to obtain a phase difference distribution matrix [22]. The average value of a specific row or column in this matrix is taken as the final phase difference.

Step_4: The obtained phase difference is substituted into the corresponding grating period in Equation (5) from Section 2 to calculate the displacement Δx between the two alignment marks.

To verify the feasibility and accuracy of the proposed method, a capacitive sensor was used as a reference (capacitive sensor probe: CSH05, controller: DT620, with a static resolution in the sub-nanometer range). A manually adjusted high-sensitivity displacement stage was used to move the wafer and mask alignment marks relative to each other. The capacitive sensor recorded the relative displacements of the mask with respect to the wafer in the x, y, and z directions through three separate channels.

**Figure 11:**
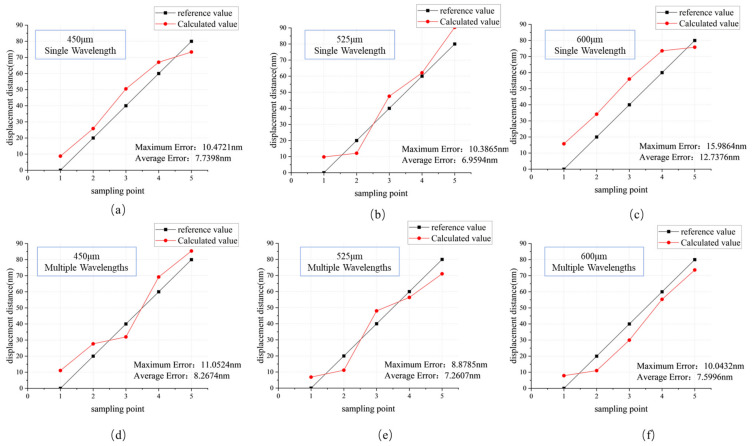
Reference displacement and calculated displacement. (**a**) Alignment error at 450 μm under traditional single wavelength, (**b**) alignment error at 525 μm under traditional single wavelength, (**c**) alignment error at 600 μm under traditional single wavelength, (**d**) alignment error at 450 μm under multiple wavelengths, (**e**) alignment error at 525 μm under multiple wavelengths, (**f**) alignment error at 600 μm under multiple wavelengths.

For each displacement measurement recorded by the capacitive sensor, the corresponding Moiré fringe-based displacement estimation was compared. Figure 11 shows the difference between the calculated offset after fringe analysis from five experiments under both multi-wavelength and single-wavelength conditions and the measured values from the capacitive sensor. In the figure, a single-wavelength alignment utilizes 532 nm green light. Since 450 μm and 525 μm fall within the DOF range of the single-wavelength alignment light source, the displacement calculation is relatively accurate. However, at 600 μm, which is within the WDF region of the 520 nm light source’s self-imaging, the contrast of the acquired Moiré fringe signal is not high enough. This results in a larger discrepancy between the calculated phase value and the actual phase after phase analysis, thereby affecting alignment accuracy. In Figure 11c, we can observe a significant decline in alignment accuracy. In Figure 11d–f, we can see that at these three positions, the multi-wavelength light source compensates for the WDF region, leading to relatively stable deviations in the calculated values. Furthermore, with the introduction of multi-wavelength alignment, the calculated deviations are nearly the same as those obtained under single-wavelength alignment, without a significant decline.

These errors mainly originate from fabrication inaccuracies of the alignment marks on the mask and wafer, image acquisition errors due to the CCD pixel size, phase extraction algorithm errors, manual displacement stage movement errors, and environmental disturbances.

Firstly, during the fabrication of alignment marks, limitations in processing precision can result in an uneven grating surface, tilted sidewalls, and non-uniform etching, all of which affect the diffraction efficiency of the grating and ultimately degrade the quality of the Moiré fringes. Secondly, since both the CCD pixel size and the grating linewidth are in the micrometer range, image acquisition is affected by truncation errors, further amplifying the non-uniformity of the grating linewidth.

Furthermore, in image processing and phase extraction, the discretization of sampled points converts continuous signals into digital signals, inevitably introducing errors that amplify alignment offsets. The manually adjusted displacement stage has a precision in the micrometer range, meaning there is a discrepancy between the actual displacement and the scale readings. Finally, as the experiment was conducted on a standard optical platform, environmental disturbances can affect the piezoelectric ceramics, inevitably causing random errors during the wafer’s movement.

## 5. Conclusions

In summary, we propose an alignment scheme based on multi-wavelength Moiré fringe phase analysis using a dual-layer grating. The scheme explores and verifies the Talbot self-imaging effect under different wavelengths while extending the alignment system’s depth of focus by utilizing the principle of optical field superposition under multi-wavelength conditions. Additionally, the frequency-doubling effect of Talbot self-imaging under multi-wavelength conditions is employed to reduce the processing difficulty of alignment markers, and its feasibility is verified. Furthermore, extensive experimental verification is provided regarding the improvement of Moiré fringe contrast after extending the depth of focus. The paper also offers a detailed analysis of the alignment accuracy and error sources of Moiré fringes under multi-wavelength conditions. Theoretical analysis and experimental results show that, within a working distance of 1 mm, the achievable depth of focus exceeds 400 μm, and the alignment accuracy remains at the nanometer level.

## Figures and Tables

**Table 1:** Contrast evaluation metrics at different wavelengths and distances.

	Indicators	532 ± 10 nm 633 ± 10 nm	532 ± 10 nm	633 ± 10 nm
Distance				
Z = 450 μm		$C_{Weber}=$ 0.5290 $C_{M}=0.9273$ $C_{RMS}=$ 54.2377	$C_{Weber}=0.4310$ $C_{M}=0.9251$ $C_{RMS}=32.2558$	$C_{Weber}=0.4820$ $C_{M}=$ 0.9623 $C_{RMS}=17.9848$
Z = 600 μm		$C_{Weber}=$ 0.5026 $C_{M}=0.9173$ $C_{RMS}=$ 51.8311	$C_{Weber}=0.4138$ $C_{M}=$ 0.9333 $C_{RMS}=33.2524$	$C_{Weber}=0.4814$ $C_{M}=0.9216$ $C_{RMS}=18.9990$

In the table, the best performance for each contrast evaluation metric is underlined, and the sec-ond-best is bolded.

## Data Availability

The data underlying the results presented in this paper are not publicly available at this time but may be obtained from the authors upon reasonable request.
